# Processing of Superfine Grinding Corn Straw Fiber-Reinforced Starch Film and the Enhancement on Its Mechanical Properties

**DOI:** 10.3390/polym10080855

**Published:** 2018-08-02

**Authors:** Min Wu, Fei Gao, Dong-Min Yin, Qi Luo, Zong-Qiang Fu, Yu-Guang Zhou

**Affiliations:** 1 College of Engineering, China Agricultural University, No. 17 Qinghua East Road, Haidian District, Beijing 100083, China; minwu@cau.edu.cn (M.W.); sophia.gophi@gmail.com (F.G.); yindongmin@cau.edu.cn (D.-M.Y.); qiluo2128@126.com (Q.L.); 2 School of Materials Science and Mechanical Engineering, Beijing Technology and Business University, No. 11 Fucheng Road, Haidian District, Beijing 100048, China; fzqxiaoqiang@163.com; 3 Key Laboratory of Clean Production and Utilization of Renewable Energy, Ministry of Agriculture and Rural Affairs, Beijing 100083, China; 4 National Center for International Research of BioEnergy Science and Technology, Ministry of Science and Technology, Beijing 100083, China

**Keywords:** corn straw, superfine grinding, starch-based film, mechanical properties

## Abstract

In this study, corn straw (CS) was reduced in size using the superfine grinding process to generate powders with particles of varying sizes (9~16 μm). The lignin, hemicellulose, and cellulose content; particle size distribution; and scanning electron microscopy (SEM) of the CS samples were analyzed. Superfine CS, of varying particle sizes, was added to the starch-based films (SF) in various amounts. The resulting corn straw starch-based films (CS/SFs) appeared to have significantly different properties, compared to the original starch-based film (SF, *p <* 0.05). The power law model and Burger’s model were used to investigate the dynamic mechanical analysis, which indicated that the mechanical properties of CS/SF performed better than that of SF, especially CS/SFs at 0.5–1.5 h ball milling and CS/SFs at a 15% addition amount. The power law model and Burger’s model also presented a strong correlation with the experimental data (>0.90).

## 1. Introduction

China produces more than 300 million tons of corn stalk each year. Currently, the main utilization methods include direct field return, pellet compression, feed addition, biomass energy, and so on [1]. The comprehensive utilization rate is less than 40%. A large amount of straw incineration and random disposal has a serious impact on soil diseases [2,3], so it is important to promote a new approach to solving the problems. Mulching soil with plastic film is a well-established practice and has served as a protective measure that is widely applied in most areas of China [4]. The benefits of plastic mulch film are that it reduces water loss, regulates soil temperature, improves the microclimate around a plant, and increases crop yield [5,6]. However, the sustainability of agro-ecosystems has been impaired by the utilization of plastic films; soil microorganisms have difficulty degrading these materials, contributing to soil compaction and loss of soil organic matter [7]. Therefore, the development of biodegradable materials that alleviate the lack of plastic biodegradation has attracted scientific interest.

Starch and starch-based materials are implemented in papermaking, textiles, pharmaceutical, and biological materials because of their inherent biodegradability, low cost, non-toxicity, and film-forming capability [8,9]. Yet, the use of thermoplastic starch is limited in industrial applications, owing to its weak resistance against mechanical stress, high susceptibility to thermal decomposition, and decreased water vapor and oxygen transmission rates [10,11]. Starch is usually incorporated into materials by first blending with hydrophobic polymers, which act as plasticizers, to improve the performance of the product and increase the range of application. Plasticizers form hydrogen bonds with starch, replacing the strong interaction among hydroxyl groups of starch molecules, which could make starch display plasticization [12,13,14].

Cellulose is a complex carbohydrate that can be obtained from natural fibers through physical or chemical treatments; the resulting material displays excellent mechanical properties [12,15]. Agricultural residues, such as straw, contain cellulose, hemicellulose, and lignin, in which cellulose networks are deeply embedded in hemicelluloses and lignin matrices. This subsequently impedes the utilization of cellulosic raw materials [16,17]. Thus, straw has to be pretreated prior to its utilization. Superfine grinding technology is a mechanical pretreatment that results in a material with a reduced particle size, destroyed cellulose, hemicellulose and lignin supramolecular structures, and decreased binding of internal microstructures [18]. Many reports have previously documented that superfine grinding is a powerful approach for breaking fiber intermolecular hydrogen bonds and for significantly increasing cellulose amorphous contents [19,20]. In addition, some research has stated that cellulose fiber and starch with appropriate plasticizer can show better mechanical properties in the food packaging industry [21]; other authors have shown that structure, properties, and the possibility of starch-based materials have been influenced by the amylose/amylopectin ratio [22].

Numerous studies have used cellulose nanowhiskers/cellulose nanofibrils or microcrystalline cellulose to strengthen and modify starch membranes [23]. The process and preparation conditions are complex. To consider the practical applications, we propose the physical processing method (ball milling) to directly process the raw corn straw materials, which could simplify the process, and reduce secondary pollution. We believe that it is a new micro-particle modification and application idea of corn straw.

In this study, superfine grinding techniques, with variations in processing times, and solids loading were used as a pretreatment to produce corn straw (CS) with particle sizes ranging from ~9 μm to ~16 μm. The physical and chemical properties of the CS samples were used to optimize the particle size and to determine the amounts needed to be added to starch-based films (SF). The CS/SF were tested by dynamic mechanical analysis (DMA).

## 2. Materials and Methods

### 2.1. Raw Materials

The corn straw (CS) was collected from Henan Province, China, in 2016. The corn straw was air dried to a moisture content of (7.2 ± 0.1)%. The corn starch was obtained from Yujing Food Co., Ltd. (Zhengzhou, Henan, China). The xylitol was purchased from Tianjin Silicon Valley Technology Development Co., Ltd. (Tianjin, China). The glycerol was secured from Beijing Chemical Company (Beijing, China). The purchased chemicals were of analytical grade and were utilized without further purification. Deionized water was used in all of the experiments.

### 2.2. Preparation of Corn Straw Samples with Different Particle Sizes

The raw CS was cut to 3 to 5 cm in length, using a hay cutter; the resulting material was subsequently coarsely milled with a grinding machine (RT-34, Rongcong Co., Ltd., Taipei, Taiwan); and the resulting material was sieved through a 1.00-mm screen and was denoted as CS-0h. A superfine vibration ball mill (CJM-SY-B, Taiji Ring Nano Products Co., Ltd., Qinhuangdao, Hebei, China) was used to produce samples in different particle sizes. This was done by mixing CS-0h and ZrO_2_ balls (6 to 10 mm in diameter) in a volume ratio of 1:2 for 0.5, 1, 1.5, 2, 4, and 8 h, respectively. The samples obtained by different grinding durations were denoted as CS-0.5h, CS-1h, CS-1.5h, CS-2h, CS-4h, and CS-8h, respectively. A water cooling circulation system served to control the grinding process; the temperature did not exceed 20 °C.

### 2.3. Determination of Structural Carbohydrate Contents

The cellulose, hemicellulose, and lignin of corn straw were determined according to the NREL/TP-510-42618 [24] and ASTM E1721-01 analytical methods [25]. The ash of the corn straw was determined according to the ASTM E1755-01 method [26].

### 2.4. Particle Size Distribution

A laser diffraction particle analyzer (Mastersizer 3000, Malvern Co., Ltd., Malvern, UK) was used to measure the particle size distribution of the CS before and after pretreatment. Air was used as a dispersing medium.

The mean particle sizes, D_10_, D_50_, and D_90_, were determined using particle size distribution curves, and corresponded to the 10th, 50th, and 90th percentiles, respectively, of the total volumes. To evaluate the overall heterogeneity of the particle size of the CS powders, the span was calculated as (D_90_ − D_10_)/D_50_ [27].

### 2.5. Scanning Electron Microscopy (SEM)

The CS samples were dried at 105 °C for 8 h and then sputtered with platinum for 2 min, using an Iron Sputtering System (Hitachi E-1010, Hitachi Co., Ltd., Tokyo, Japan). The shape and the surface characteristics of the CS samples were determined with a Scanning Electron Microscope (Hitachi S-3400N, Hitachi Co., Ltd., Tokyo, Japan) operated at a voltage of 15 kV.

### 2.6. Preparation of CS/SF Obtained with Different Superfine Grinding Periods or with Variations in Loading

The CS/SF were prepared using the solution casting method, as previously reported by Fu et al. [28] and Li et al. [15]. The first series of CS/SF samples were prepared utilizing the following methods. A total solid of 20 g (corn starch as the main ingredient, 13.79 g; CS fiber, 2.07 g equal to 15% (*w*/*w*) of corn starch; plasticizers (glycerol: xylitol ratio of 1:1), 4.14 g equal to 30% (*w*/*w*) of corn starch) with 200 mL of deionized water were mixed to form a starch-plasticized fiber suspension with a 10% (*w*/*v*) solid concentration. The obtained suspension was mixed by stirring at 6000 r/min for 10 min (OS20-Pro, Dalong Xingchuang Experimental Instrument Co., Ltd., Beijing, China) and heated in a water bath (HHS-4S, Haolong Instrument Equipment Co., Ltd., Shanghai, China) at 99.9 °C for 1 h at 300 r/min, then the starch components were completely gelatinized. The water evaporation was minimized by covering the resistant films with a plastic wrap. The starch paste was cooled at atmospheric conditions for 10 min, and subjected three times to high pressure homogenization (AH 100D, ATS Engineering Inc., Shanghai, China) at 30 MPa, then, the air bubbles were finally removed from the paste.

The films were prepared by placing 15 mL of starch paste into 9 cm diameter polycarbonate petri dishes; the resulting material was dried in an electrothermal dry oven (101-3, Yiheng Scientific Instrument Co., Ltd., Shanghai, China) at 45 °C for 8 h. Afterwards, the disks were placed in a constant temperature humidity chamber (JYH-152, Jiayu Scientific Instrument Co., Ltd., Shanghai, China) at 25 °C and 43% relative humidity (RH) for one week prior to use.

Two series of CS/SF samples were prepared utilizing the following methods. The first group of CS/SF samples was prepared with CS that underwent variations in the ball grinding durations; the CS was added to make up 15% *v*/*v* of the final mixture. The CS/SF samples were designated as follows: CS/SF-0.5h, CS/SF-1h, CS/SF-1.5h, CS/SF-2h, CS/SF-4h, and CS/SF-8h, which corresponded to ball milling treatments of 0.5, 1, 1.5, 2, 4, and 8 h, respectively. CS/SF-0h was a control sample that was added to the original corn straw and prepared via the same approach.

The second group of CS/SF samples was prepared by varying the amount of CS to the mixture. The CS was of a uniform particle size and was obtained through 1 h of ball milling, because the average particle diameter of the corn straw after 1 h ball of grinding is the smallest of all of the samples. These samples were designated as CS/SF, 5%; CS/SF, 10%; CS/SF, 15%; and CS/SF, 20%, which corresponded to additions of 5%, 10%, 15%, and 20% of CS, respectively. The pure SFs that did not contain CS were prepared in the same way and were used as the control sample.

### 2.7. Measurement of the Basic Properties of CS/SF

The thicknesses of the films was measured using a digital micrometer (Mitutoyo Co., Tokyo, Japan) at ten random points in each film. The film density was determined according to the method reported by Dias et al. [29]. The moisture content was measured using a drying oven method [30], and was calculated from the weight loss and expressed as a percentage. The rectangular films were dried at 105 °C for 24 h using a drying oven (101-3, Yiheng Scientific Instrument Co., Ltd., Shanghai, China). The film solubility was determined according to the method described by Arfat et al. [31]. The film transparency was measured using a UV-visible spectrophotometer (Persee TU-1810, General Analysis Co., Ltd., Beijing, China), according to Arfat et al. [32]. The water vapor permeability (WVP) was conducted using the ASTM E96-00 method with some modifications, as described by Wu et al. [12].

### 2.8. Frequency Sweep Test

The temperature of the test was set at 30 °C, and the frequency ranged from 1 Hz to 50 Hz. The force track was set at 125%. The controlled strain was set at 0.1%, and the storage modulus and loss modulus of the samples were recorded to create the corresponding frequency sweep curves [32]. The frequency dependence of the storage modulus and loss modulus of the samples can be fitted by the power law model, as follows:

(1) $$G^{\prime }=K^{\prime }\cdot f^{n^{\prime }}\;$$

(2) $$G^{\prime \prime }=K^{\prime \prime }\cdot f^{n^{\prime \prime }}\;$$

where *K*′ and *K*″ are the power law parameters with units of Pa·s^n^ and rad/s, respectively; and *n*′ and *n*″ are the frequency indices, which reflect the frequency dependence of the sample elasticity modulus and viscosity modulus, respectively.

### 2.9. Creep and Creep-Recovery Measurements

A dynamic mechanical analyzer (DMA, Q800, TA Instruments, New Castle, DE, USA) was used to determine the creep and creep-recovery of CS/SF and SF (control). These films were cut into 40 mm × 7 mm rectangular strips using a film tensile clamp. One end was clamped to the fixed grip and the other end was clamped to the movable grip of the DMA furnace. Then, a small preload (0.01 N) was applied to keep each film gently stretched [33]. The force track was set at 125%. After equilibrating at 35 °C for 2 min, a stress of 1 MPa was supplied to the films for 5 min. The stress was removed and the films were recovered for 5 min. The strain and creep compliance data were recorded as a function of time.

### 2.10. Burger’s Model

According to the data from the creep and creep-recovery test, Burger’s model was calculated as follows [34]:

Creep equation of the Burger’s model is as follows:

(3) $$\varepsilon (t)=\frac{\sigma_{0}}{E_{M}}+\frac{\sigma_{0}}{E_{K}}(1-e^{-t/\tau })+\frac{\sigma_{0}}{\eta_{M}}\cdot t\;$$

(4) $$\tau =\frac{\eta_{K}}{E_{K}}\;$$

Based on the Burger’s model, time (*t*) was derived and then the creep rate was calculated.

(5) $$\varepsilon^{\prime }(t)=\frac{d\varepsilon (t)}{dt}=\frac{\sigma_{0}}{\eta_{k}}e^{-t/\tau }+\frac{\sigma_{0}}{\eta_{M}}\;$$

When the creep time tends to infinity, the creep rate will gradually stabilize and eventually become a fixed value.

(6) $$\varepsilon^{\prime }(\infty )=\frac{d\varepsilon (t)}{dt}{|}_{t=\infty }=\frac{\sigma_{0}}{\eta_{M}}\;$$

where *ε_t_* (dimensionless) represents the creep strain of a film; *t* denotes the time (s) after loading; *σ_0_* represents the loaded stress, 1 MPa; *E_M_* and *η_M_* represent the modulus and viscosity of the Maxwell spring and dashpot, respectively; *τ* = *η_K_*/*E_K_*, is the retardation time taken to produce 63.2% (1 − e^−1^) of the total deformation in the Kelvin unit; and *E_K_* and *η_K_* represent the modulus and viscosity of the Kelvin spring and dashpot, respectively. The experimental *ε_t_* versus *t* data were fitted to Equation (1) with SPSS 21.0 (SPSS Inc., Chicago, IL, USA), and the four parameters (*E_M_*, *E_K_*, *η_M_*, and *η_K_*) were obtained.

### 2.11. Statistical Analysis

All of the experiments were carried out in triplicate. The experimental DMA data was obtained directly from the Universal Analysis 2000 data analysis software (TA Instruments, New Castle, DE, USA). The values were expressed as mean ± standard deviation. Duncan’s multiple comparison tests were employed to determine the significant difference among the mean values using SPSS 21.0 software (SPSS Inc., Chicago, IL, USA) at the confidence level of 95% (*p* < 0.05).

## 3. Results and Discussion

### 3.1. The Basic Properties of the Corn Straws with Different Particle Sizes

#### 3.1.1. The Constituent Contents of the Corn Straws with Different Particle Sizes

The cellulose, hemicellulose, and lignin contents of the CS samples that underwent various ball milling treatments are presented in Table 1. Compared to the control samples (CS-0h), the cellulose and hemicellulose contents of the samples with ball grinding significantly increased (*p* < 0.05). Increasing the ball grinding times resulted in significant differences in the cellulose and hemicellulose contents (*p* < 0.05), which increased first and then decreased. However, the opposite trend was presented in the contents of the lignin and ash, which were consistent with a recent study of Ji et al. [18]. The cellulose and hemicellulose contents in the samples increased and the lignin contents declined, which may have been caused by the lignin structure being effectively destroyed by the superfine grinding. After 2 h of ball grinding, the contents of the cellulose, hemicellulose, and lignin increased again, which might be due to the superfine particles being produced by the long ball grinding duration, which contributed to the aggregation phenomena of the particles.

The cellulose content increases to a certain extent with the increase of ball milling time, in which the maximum value is reached at the ball milling time of 1 h. This may be due to the shearing action of the ball milling to break the straw structure and precipitate cellulose. The hemicellulose content increased significantly with the milling time, which may be due to the shear action of the ball mill, which caused the macromolecular polysaccharide structure of the cellulose to degrade and break into hemicellulose [35,36]. The lignin structure is relatively stable, and its effect on ball milling is not significant.

The aggregation of the CS powder can be attributed to material crushing procedures, in which the CS particles absorb mechanical and thermal energies, resulting in increases of their surface energy that favors particle cohesiveness. Electrostatic Coulomb forces were produced by positive and negative charges that are on the surfaces of the superfine powders. The distance between the particles was reduced when the material was refined to a certain particle size. Additionally, the van der Waals force between particles, being greater than their respective gravity, resulted in a hydrogen bond or other chemical bonds, increasing the cohesiveness.

#### 3.1.2. Morphological Properties of CS of Various Particle Sizes

**Figure 1:**
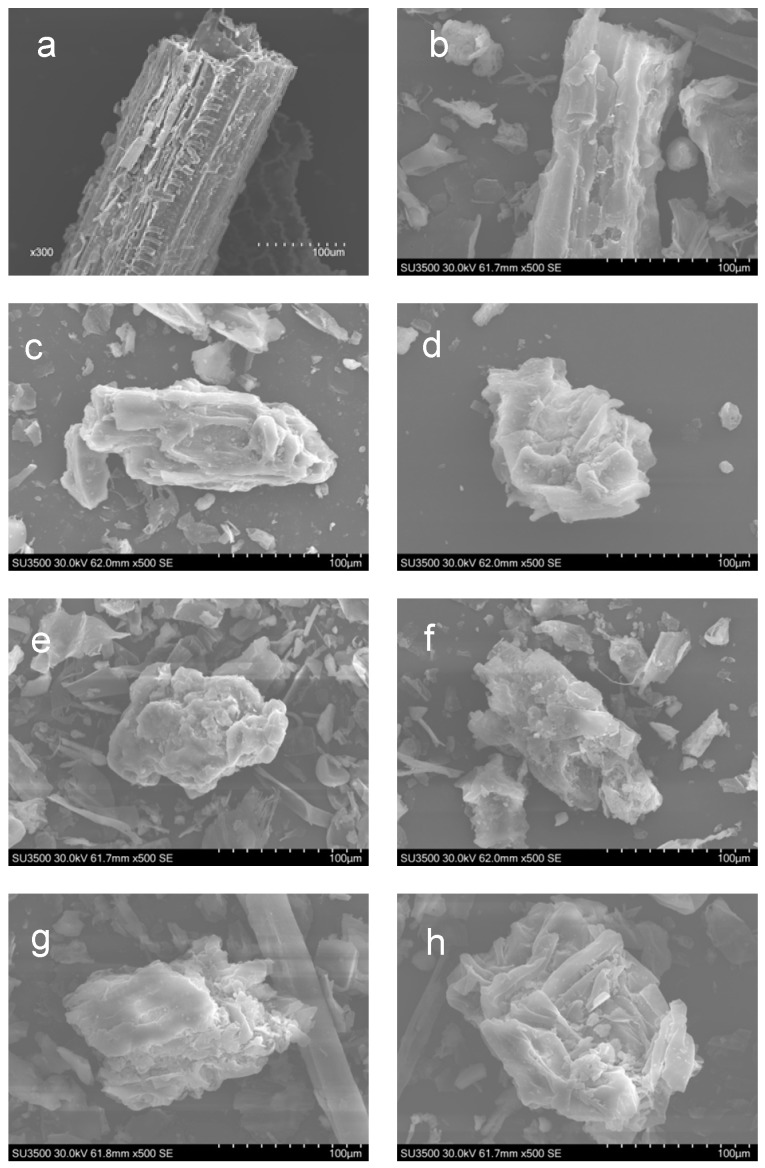
SEM images of the corn straws (CSs) with different ball grinding times ((**a**,**b**): CS-0h; (**c**): CS-0.5h; (**d**): CS-1h; (**e**): CS-1.5h; (**f**): CS-2h; (**g**): CS-4h; and (**h**): CS-8h).

Superfine grinding destroys the structure of the vascular bundles and releases fibrous yarns; the resulting material could be useful for connecting and supporting the starch-based films. Scanning electron microscopy (SEM) was used to examine the surface microstructure of the CS powder particles, as shown in Figure 1.

As presented in Figure 1a, sheet-like fibrous structures were observed in the control group (CS-0h). Tissue and fibrous structures were intact in the CS that did not undergo superfine crushing; only a small number of epidermal cells were destroyed. In the CS-1h material, the sheet-like structures were destroyed, in which the material was fragmented into short-chain and irregular fibrous structures, as shown in Figure 1c. This accorded well with the results of the particle size distribution. The CS-0.5h and CS-1h samples displayed shorter lengths of corn straw particles. The CS material that was subjected to crushing times greater than 1 h, CS-1.5h to CS-8h were agglomerated; the particle surfaces became rougher and attraction between the particles increased. This was particularly noticeable with samples of CS-4h and CS-8h. The increased agglomeration was related to the decrease of the cellulose, hemicellulose, and lignin content of the CS with different particle sizes.

#### 3.1.3. Particle Size Distribution of CS

The average particle diameters and spans of the CS samples with different particle sizes are presented in Table 2. Compared with the control sample (CS-0h), the particle sizes of the superfine crushing samples were reduced; the average particle size was approximately 10 μm. The CS-1h material presented the lowest particle diameter (~9 μm, *p* < 0.05), indicating that the processing times below 1 h result in crushing rather than grinding. As the processing times were increased, the CS particle sizes were larger and could be attributed to the increasing amount of the surface activity by the longer mechanical fragmentation, as previously reported [36]. The spans of corn straws with different particle sizes revealed an increasing trend during the first 1.5 h of the grinding process and tended to decrease subsequently, which agreed with the results reported by Ji et al. [18].

The *S_SPS_* value of the powders is an indicator of the particle size distribution. Smaller *S_SPS_* values are representative of narrower distributions, in which only a few particles were either bigger or smaller than the mean. As shown in Table 2, the *S_SPS_* values were the greatest for the CS samples CS-0.5h, CS-1h, and CS-1.5h. With ball grinding times greater than 1.5 h, the *S_SPS_* value decreased and this could be mostly due to the particle size becoming homogeneous during treatment.

### 3.2. Properties of the Starch-Based Films Blended with CS

The thickness, density, moisture content, solubility, transparency, and WVP of the starch-based films blended with the CS displaying various physical properties are presented in Table 3. Overall, the thickness of the CS/SFs was somewhat constant with values of 0.20 ± 0.01 mm, which provided an ideal foundation for the subsequent determination of the film properties. Compared to the density of the control SF material, that of constant CS ratio CS/SF material increased significantly (*p* < 0.05) as a function of the material produced as a result of the increased grinding time. The increase in density could possibly be attributed to the reduction in the CS particle diameter (*p* < 0.05) as a function of the increased grinding time. The reduction in the CS particle diameter was substantiated by SEM images. The reduced CS particles could possibly have an increased number of linkages with the branched chain structure of starch, resulting in the formation of compact films with an enhanced performance. Specifically, the CS/SF films displayed increased densities when the time of the superfine grinding exceeded 2 h, which was probably due to the agglomeration phenomenon of the corn straw sample. There is good interfacial adhesion between the starch and cellulose because both contained hydroxyl groups, which can form hydrogen bonds between the interfaces [37]. In addition, the stability performance of the starch-based films with corn straw can be attributed to the higher dispersion of the nano- or micro-particles in the matrix of the film, due to the higher hydrogen bridges with the OH groups of the starch [38].

The moisture content of the SF material is related to the void volume of the water molecules, and is also an important parameter that affects the biofilm properties [39]. The solubility of the film material in the water was characterized by the hydrophilicity of the film, which benefited the soil mulch property when the solubility was negligible. Table 3 presents the results of the moisture content solubility of CS. Compared with the control SF, and prepared at a similar CS ratio, the moisture content and solubility of CS/SF-0.5h, CS/SF-1h, and CS/SF-1.5h were significantly reduced (*p* < 0.05). This can be attributed to the fact that the water affinity of the CS/SF films, prepared with a CS material that has been ground for 0.5, 1, or 1.5 h, was significantly reduced (*p* < 0.05), and to the increased time required for complete degradation of the starch-based films.

In general, the greater transparency values correspond to a lower transparency of th efilms. As seen in Table 3, the transparency of the CS/SF films was significantly lower than the CS/SF-0h. With the increased grinding times, the transparency of the CS/SF materials declined, most likely due to the reduction of the CS particle size, resulting in augmented linkages with starch molecules. The WVP values for the CS/SF films produced with CS obtained at various grinding times are presented in Table 3. Compared with the control sample, CS/SF-0h, 0.53 gm^−2^·h^−1^·Pa^−1^, the WVP values of the CS/SF films were significantly reduced (*p* < 0.05). The CS/SF-2h presented the lowest WVP value of 0.29 gm^−2^·h^−1^·Pa^−1^. Increasing the superfine grinding time resulted in increased WVP values. The WVP values of the materials are closely related to their network structure, in which the lower WVP values correspond to denser film structures. Without superfine grinding, the resulting straw surfaces were rough, impeding mixing with starch molecules and reducing the film attributes. Meanwhile, after a relatively short period of time (e.g., ~1 h) of superfine grinding, the straw particle size decreased sufficiently, allowing for the assembly of a denser network structure.

Table 3 presents values for thickness, density, moisture content, solubility, transparency, and the WVP of CS/SF films as a function of the changes in the CS ratio. Compared to the thickness of the control, augmenting the proportion of the CS significantly increased density, moisture content, and solubility (all *p* < 0.05). The transparency values of these CS/SF films were greater than that of the control, as shown in Table 3. On the other hand, Table 3 lists the WVP values of the CS/SF films prepared with various CS ratios; they were significantly lower than that of the control sample (SFs, *p* < 0.05). With increases in the amount of CS being added, the WVP value of the CS/SF varied.

### 3.3. Frequency Sweep Properties of the CS/SF Films with Different Particle Sizes or Addition Amounts

**Figure 2:**
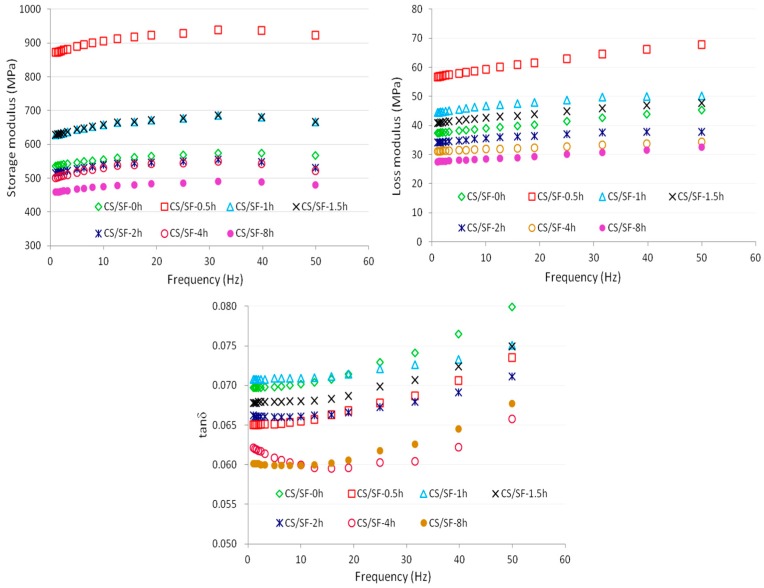
Storage modulus, loss modulus, and tan *δ* versus frequency curves of the corn straw starch-based films (CS/SFs) with different ball grinding times.

**Figure 3:**
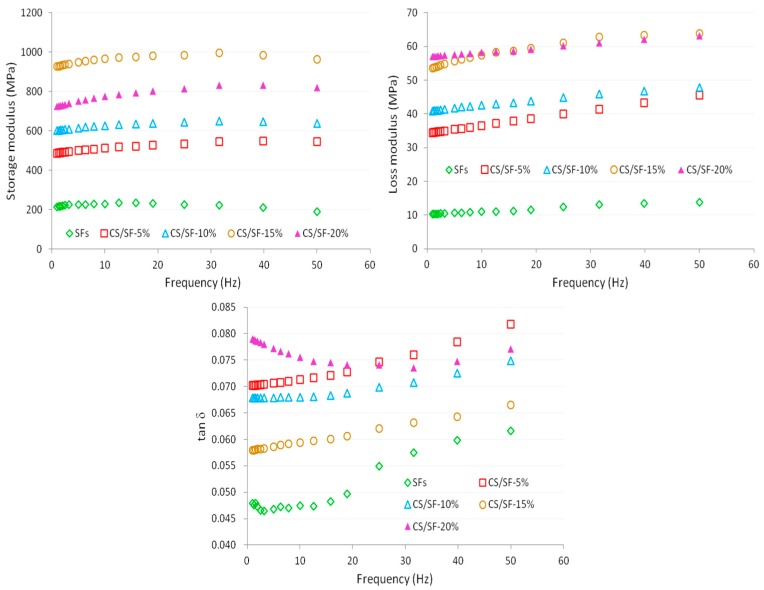
Storage modulus, loss modulus, and tan *δ* versus frequency curves of the CS/SFs with different addition amounts.

The storage modulus (*G*′), loss modulus (*G*″), and tan δ of the CS/SFs with different ball grinding times as a function of frequency are listed in Figure 2. The loss modulus (*G*″) and tan δ of the CS/SFs increased as the frequency increased; however, the storage modulus (*G*′) slightly decreased after the increase. This may be due to the fact that as the frequency increases to 40–50 Hz, the loading speed changes faster, which leads to the elastic relax of the film not being completed in a short time; then, a small amount of energy is absorbed by the film, that is, elastic hysteresis occurs. A similar tendency is also found in Figure 3, which illustrates the storage modulus (*G*′), loss modulus (*G*″), and tan δ of the CS/SFs with different addition amounts as a function of the frequency. The values of *G*′ are much higher than those of *G*″ in the entire frequency range, which indicates that the CS/SFs showed dominant elastic behavior compared to viscous behavior [39].

As can be seen from Figure 2, the storage modulus (*G*′), loss modulus (*G*″), and tan δ tend to increase first and then decrease with the addition of CS at different ball milling times. When adding the CS-0.5h, the maximum values of *G*′ and *G*″ of the CS/SFs are reached. This illustrated that adding the CSs at a suitable ball milling time (0.5–1.5 h) can improve the mechanical properties of the starch-based film material, and CS/SFs are also affected by the interfacial stress of the matrix and the filler [40]. Ball milling can reduce the particle size of CS; however, the long ball milling time could produce a large amount of electrostatic agglomeration in the CS, which leads to an uneven distribution in the starch matrix, and affects the cross-linking effect with the starch interface. Hence, the obtained CS/SF material has poor mechanical properties.

In Figure 3, *G*′, *G*″, and tan *δ* first increase and then decrease with the increase of the proportion of CS fiber. The results demonstrate that adding the CS to the ball mill process could significantly improve the mechanical properties of the film material, which is due to the hydrogen bond between the CS and starch [41]. The internal micro or nanoscale structures can improve the functional properties and stability of the starch-based films, and promote strong interactions between them [42].

The power law model was chosen to predict the frequency dependence of *G*′ and *G*″ by using Equations (1) and (2) [43]. The values of *K*′, *n*′, *K*″, and *n*″ are listed in Table 4. It can be seen that the regression coefficients of the *G*′ versus *f* data are around 0.90–0.99, which demonstrated that the power law model is suitable for the prediction of the experimental *G*′ versus *f* data. In CS/SFs at different ball grinding times, CS/SF-0.5h and CS/SF-1h present higher *K*′ and *K*″ values than the CS/SFs; in the CS/SFs with different addition amounts, the CS/SFs show significantly higher *K*′ and *K*″ values compared to SFs (*p* < 0.5), especially CS/SF-15%, having the highest *K*′ value. The observations indicate that the CS/SFs are more elastic or solid-like compared to the SFs, and the presence of CS could enhance the elastic component of the starch-based films.

### 3.4. Dynamic Mechanical Properties of the CS/SF Films with Different Particle Sizes or Addition Amounts

**Figure 4:**
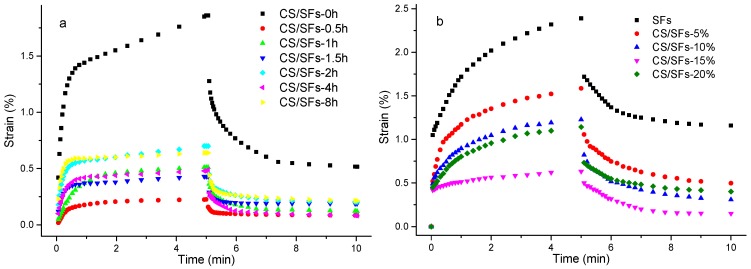
Creep-recovery versus time curves of the CS/SF with different ball grinding times (**a**) and different addition amounts (**b**).

The short-term creeping and creep-recovery strains as a function of time of the CS/SF films are presented in Figure 4a. The strain curves for all of the samples show a similar trend over time, that the instantaneous strain rose sharply, flattened, and levelled out. During the recovery phase, the curves displayed typical creeping behavior. When the material was subjected to external forces, creep occurred, indicating that the viscoelastic property of the material was decreasing [44]. The creeping strain, irreversible strain, and creep compliance of CS/SF were significantly lower than that of the control SF, indicating that the addition of superfine pulverized CS enhanced the creep resistance of the SFs. CS/SF-0.5h presented the lowest creep strain and creep compliance, only 15.4% of that of SF, and this demonstrates that the superfine crushing straw fiber can be well distributed into starch films, decreasing the mobility of the starch chains and improving resistance. The creeping and irreversible strains of CS/SF films increased; this may be due to the long grinding times that may have triggered aggregation, contributing to uniform dispersal throughout the starch matrix.

In the recovery stage, the irreversible deformations of the CS/SF exhibited similar behavior as during the creeping phase, most likely due to the viscous nature of the SFs. The creep-recovery results of the CS/SF-time films indicated that the short-time superfine grinding preparation proved to be conducive to the improved mechanical properties of the CS/SF.

The short-term creep and creep-recovery strains as a function of adding SFs and CS/SF are displayed in Figure 4b, which indicates that the strain curves of all of the sample films showed typical creep characteristics. After adding the CS powders, the creep strain, irreversible deformation, and creep compliance of the CS/SFs were significantly lower than those of SFs, indicating that straw fiber weakened the creep property of starch film but enhanced their creep resistance. When the amount of straw fiber was less than 15%, the creep deformation of the CS/SF decreased as more CS was added. Once the added amount of 15% was reached, the creep strain of CS/SF showed the smallest value, which was equivalent to 32% of the SFs. This indicated that the straw fiber was well distributed in the starch films. When the starch films were subjected to external forces, the straw fiber impeded the movement of the starch chains, enabling the SF to sustain a greater external force. However, when the added amount of straw exceeded 20%, the creep strain and irreversible deformation of the CS/SF increased. The probable cause of this was, that when the addition of straw fiber reached a certain level, aggregation of the straw fiber occurred and could not be well distributed, affecting the appearance of the sample.

During the recovery stage, the irreversible deformation of the CS/SFs exhibited the same pattern as in the creep stage, due to the viscous nature of the starch films. The results of the creep-recovery illustrated that the CS/SF with a 15% addition had a higher viscoelasticity and greater creep resistance ability. When the added amount was less than 15%, the mechanical properties of the CS/SFs improved to varying degrees.

### 3.5. Burger’s Model Analysis

Burger’s model (Equation (3)), composed of the Maxwell and Kelvin–Voigt models, is widely used to characterize the viscoelastic properties of polymer materials [15,45]. Burger’s model consists of three essential parts. The first part represents the immediate elastic deformation caused by the change in bond length and the bond angle within the molecule (the first item on the right side of Equation (3)), which is a constant value that does not change with time. The second part indicates the delayed elastic strain caused by the stretch and curl of the segment (the second item on the right side of Equation (3)), which shows the earliest stage of creep and attains a saturation value during a relatively short period of time. The third part shows the viscous flow caused by the slip between the polymers (the last item on the right side of Equation (3)), characterizing the long-term creep trend of the polymer and changing linearly with time. Therefore, the amount of deformation of the polymer is always equal to the sum of these three parts, which also confirms that the deformation of the polymer has both elastic and viscous properties.

The values of the five parameters (*E_M_*, *E_K_*, *η_M_*, *τ*, and *ε*′(*∞*)) and the coefficient of determination *R*^2^ of Burger’s model for the creep-recovery test of the CS/SF produced from different superfine grinding times are presented in Table 5. Burger’s model showed a satisfactory agreement with the experimental strain versus time data (*R*^2^ > 0.950).

*E_M_* characterizes the modulus of elasticity of the Maxwell model, which can cause instantaneous creep deformation but can be recovered immediately after the removal of stress. The larger the *E_M_* value, the better the material’s elasticity [15]. As can be seen from Table 5, the *E_M_* values of the CS/SF-time were significantly increased (*p* < 0.05) compared with CS/SF-0h, indicating that the CS/SF has better elasticity properties. CS/SF-1h presents the highest elastic value (*E_M_* = 16.120 × 10^8^ MPa). These results indicated that the incorporation of corn straw fiber could improve the elasticity of the starch-based films.

*E_K_* expresses the modulus of the elasticity of the Kelvin model, which represents the short-term retardant elastic deformation, that is, the stiffness of the amorphous polymer chains [46]. The ratio of *η_K_*/*E_K_* is the retardation time, *τ*, representing the relaxation time of the sample, which is the time required for the stress to decrease to an initial value of 1 − e^−1^ (about 63.2%) during the continuous deformation [47]. Therefore, the larger the *E_K_* and *τ* values are, the better the viscosity of the material will be. From Table 5, the *E_K_* value and *τ* value of the CS/SF-time increased significantly at first (*p* < 0.05) and then decreased, indicating the following: firstly, that the addition of corn straw fiber could enhance the viscosity of starch-based films, and secondly, excessive straw fiber could destroy the viscosity of starch-based films.

*η_M_* characterizes the irreversible deformation of the material. In other words, permanent deformation may be caused by the structure’s destruction of the crystalline region or the structural rearrangement of the amorphous region in the polymer. Alternatively, it might be caused by the irreversible deformation of the amorphous region. From Table 5, the *η_M_* of the CS/SF-time was significantly increased (*p* < 0.05) compared with CS/SF-0h, indicating that the micronized straw fiber weakened the viscous mobility of the starch-based films and reduced the irreversible deformation. Similarly, the CS/SF-1h showed the maximum *η_M_* value of 1607.628 ± 9.522 MPa·s^−1^, which means that the CS/SF-1h has better viscoelasticity and is more suitable for the requirements of soil mulch.

After a sufficiently long time, the creep rate will gradually attain a constant value in the steady creep stage (*t* = ∞), as shown by Equation (6). The *ε*′(*∞*) represents the long-term creep properties of the material. From Table 5, the *ε*′(*∞*) values of the CS/SF-time are significantly smaller than those of CS/SF-0h, and the CS/SF-1h is the smallest. The results demonstrated that adding corn straw could improve the internal structure of the starch-based films and modify the long-term creep resistance. Based on the above, using Burger’s model to describe the creep characteristics of the CS/SFs presents the highest degree of fit. The micronized pretreated of corn straw can effectively improve the viscoelasticity and creep resistance of the starch-based films.

The model-fitting parameters of the creep-recovery test of the CS/SF-addition are shown in Table 5. It is evident that Burger’s model can be applied for CS/SF-addition (*R*^2^ > 0.98). Following the addition of the superfine pulverized straw fiber, the *E_M_* value of the CS/SF-addition significantly increased (*p* < 0.05) compared to the SFs, indicating that the CS/SF had superior elastic properties. It is noteworthy that the CS/SF-15% presented the highest elastic value (*E_M_* = 1.752 × 10^3^ MPa). The values of *E_K_* and *τ* increased significantly (*p* < 0.05), which indicated that the addition of corn straw fiber could enhance the viscosity of the starch-based films. There was no significant difference in the retardation time between the CS/SF-addition and the SFs (*p* > 0.05), which may be due to the relatively weak influence of a change in the addition to the viscosity of the starch-based films.

In addition, the *η_M_* of CS/SF increased significantly (*p* < 0.05), indicating that the addition of straw fiber could weaken the viscous fluidity of the starch-based films and decrease the irreversible deformation. Similarly, the maximum value of *η_M_* (4.281 × 10^6^ MPa·s) was obtained for the CS/SF blended with the addition of 15%. This means that the CS/SF-15% present the smallest irreversible deformation and the best viscoelasticity, and are more suitable for the requirements of soil mulch. Table 5 shows that the *ε*′(*∞*) value of the CS/SF-addition is obviously smaller than that of the SFs.

## 4. Conclusions

In the present study, the mechanical fragmentation could effectively reduce the particle size of corn straw (9~16 μm). The corn straw prepared by superfine grinding showed better mechanical properties, such as the cellulose content and average particle diameter. The basic properties of the starch-based films blended with corn straw showed significant differences with the starch-based films (*p* < 0.05). The CS/SF films performed significantly better in terms of creep resistance properties, as compared to the starch-based films. Burger’s model described the mechanical properties of the CS/SF films well, in which *R*^2^ was greater than 0.90. The incidence of the creeping strain and the irreversible deformations of the CS/SF films were distinctly less than those of the starch-based films. The results showed that CS/SF-1h and CS/SF-15% had the best mechanical properties among all of the CS/SF films produced, according to the frequency sweep and the creep-recovery test, which confirmed that the starch-based films blended with CS fiber could be used for soil mulch instead of plastic films.

## Figures and Tables

**Table 1:** The cellulose, hemicellulose, lignin, and ash contents of the corn straw samples with different ball grinding times. CS—corn straw.

Samples	Cellulose (%)	Hemicellulose (%)	Lignin (%)	Ash (%)
CS-0h	19.94 ± 0.89 ^ab^	13.50 ± 0.73 ^a^	47.36 ± 2.07 ^abc^	5.23 ± 0.55 ^a^
CS-0.5h	19.87 ± 0.46 ^a^	14.11 ± 0.33 ^b^	49.91 ± 4.36 ^c^	6.29 ± 0.16 ^bc^
CS-1h	20.83 ± 0.58 ^c^	16.04 ± 0.98 ^d^	46.11 ± 1.04 ^abc^	5.39 ± 0.41 ^ab^
CS-1.5h	20.65 ± 0.44 ^ab^	15.87 ± 0.33 ^d^	44.07 ± 1.80 ^a^	5.97 ± 0.58 ^ab^
CS-2h	20.16 ± 0.21 ^ab^	14.95 ± 0.22 ^c^	44.83 ± 0.97 ^ab^	7.01 ± 0.37 ^c^
CS-4h	20.12 ± 0.53 ^ab^	15.22 ± 0.03 ^c^	49.04 ± 2.57 ^bc^	6.21 ± 0.42 ^bc^
CS-8h	20.73 ± 0.52 ^ab^	15.97 ± 0.09 ^d^	46.28 ± 0.45 ^abc^	5.71 ± 0.67 ^ab^

Values represent the mean ± standard deviation. ^a–^^d^ Within a row, the means with different superscript letters differ significantly (*p* < 0.05).

**Table 2:** Average particle diameters and span of corn straw samples with different ball grinding durations.

Samples	D_10_ (μm)	D_50_ (μm)	D_90_ (μm)	*S_SPS_*
CS-0h	19.17 ± 0.12 ^f^	85.33 ± 0.85 ^e^	198.00 ± 4.36 ^d^	2.10 ± 0.03 ^a^
CS-0.5h	1.98 ± 0.02 ^c^	11.50 ± 0.26 ^b^	48.10 ± 3.80 ^b^	4.01 ± 0.32 ^c^
CS-1h	1.55 ± 0.03 ^a^	9.46 ± 0.08 ^a^	43.07 ± 0.50 ^a^	4.39 ± 0.08 ^c^
CS-1.5h	1.88 ± 0.03 ^b^	11.23 ± 0.06 ^b^	51.67 ± 4.20 ^bc^	4.43 ± 0.35 ^c^
CS-2h	2.25 ± 0.02 ^d^	12.83 ± 0.21 ^c^	52.70 ± 2.23 ^bc^	3.93 ± 0.35 ^bc^
CS-4h	3.31 ± 0.08 ^e^	15.83 ± 0.32 ^d^	57.87 ± 1.15 ^c^	3.45 ± 0.03 ^b^
CS-8h	3.38 ± 0.02 ^e^	15.80 ± 0.10 ^d^	58.67 ± 0.81 ^c^	3.50 ± 0.04 ^b^

Values represent the mean ± standard deviation. ^a–^^f^ Within a row, the means with different superscript letters differ significantly (*p* < 0.05).

**Table 3:** Physical properties of starch-based films blended with corn straw of different ball grinding times and addition amounts. SF—starch-based films.

Sample	Thickness (mm)	Density (g·cm^−3^)	Moisture Content (%)	Solubility (%)	Transparency	WVP (gm^−2^·h^−1^·Pa^−1^)
CS/SF-0h	0.20 ± 0.01 ^a^	105.40 ± 0.21 ^a^	16.50 ± 0.11 ^c^	26.56 ± 0.01 ^c^	5.08 ± 0.04 ^e^	0.53 ± 0.03 ^d^
CS/SF-0.5h	0.20 ± 0.01 ^a^	124.25 ± 0.83 ^d^	11.31 ± 0.13 ^a^	22.60 ± 0.13 ^b^	2.63 ± 0.01 ^d^	0.40 ± 0.07 ^cd^
CS/SF-1h	0.20 ± 0.01 ^a^	118.07 ± 0.86 ^c^	11.38 ± 0.01 ^a^	19.78 ± 0.21 ^a^	1.15 ± 0.08 ^c^	0.33 ± 0.01 ^b^
CS/SF-1.5h	0.20 ± 0.01 ^a^	110.34 ± 0.88 ^b^	12.06 ± 0.09 ^b^	19.71 ± 0.22 ^a^	1.01 ± 0.01 ^b^	0.29 ± 0.01 ^a^
CS/SF-2h	0.26 ± 0.03 ^b^	122.44 ± 1.01 ^cd^	17.42 ± 0.21 ^d^	22.36 ± 0.08 ^b^	0.84 ± 0.01 ^a^	0.29 ± 0.01 ^a^
CS/SF-4h	0.20 ± 0.01 ^a^	130.06 ± 2.63 ^e^	29.85 ± 0.33 ^e^	26.18 ± 0.13 ^c^	0.86 ± 0.01 ^a^	0.37 ± 0.01 ^c^
CS/SF-8h	0.20 ± 0.01 ^a^	133.09 ± 5.05 ^e^	31.67 ± 0.26 ^f^	25.67 ± 0.15 ^c^	0.97 ± 0.13 ^ab^	0.34 ± 0.01 ^b^
SFs	0.18 ± 0.01 ^a^	93.40 ± 1.21 ^a^	17.50 ± 0.11 ^e^	26.96 ± 0.01 ^c^	0.18 ± 0.24 ^a^	1.02 ± 0.06 ^d^
CS/SF-5%	0.20 ± 0.01 ^a^	114.25 ± 0.83 ^b^	14.71 ± 0.13 ^d^	22.60 ± 0.13 ^bc^	0.36 ± 0.01 ^b^	0.57 ± 0.02 ^c^
CS/SF-10%	0.20 ± 0.01 ^a^	113.07 ± 0.86 ^b^	12.38 ± 0.01 ^c^	20.33 ± 0.11 ^b^	0.72 ± 0.08 ^c^	0.42 ± 0.01 ^b^
CS/SF-15%	0.20 ± 0.01 ^a^	118.07 ± 0.86 ^c^	11.38 ± 0.01 ^b^	19.78 ± 0.21 ^b^	1.15 ± 0.08 ^d^	0.33 ± 0.01 ^a^
CS/SF-20%	0.20 ± 0.03 ^a^	123.44 ± 1.01 ^d^	10.42 ± 0.21 ^a^	12.36 ± 0.08 ^a^	1.45 ± 0.01 ^d^	0.42 ± 0.01 ^b^

Values represent the mean ± standard deviation. ^a–^^e^ Within a row, means with different superscript letters differ significantly (*p* < 0.05). WVP—water vapor permeability.

**Table 4:** The parameters of power law models for CS/SFs with different ball milling times and addition amounts.

Sample	*K*′ × 10^3^ (Pa·s^n^)	*n*′	*R* ^2^	*K*″ × 10^3^ (Pa·s^n^)	*n*″	*R* ^2^
CS/SF-0h	36.085 ± 0.475 ^d^	0.045 ± 0.005 ^c^	0.922	0.532 ± 0.002 ^d^	0.019 ± 0.001 ^a^	0.952
CS/SF-0.5h	54.916 ± 0.586 ^e^	0.044 ± 0.004 ^c^	0.968	0.866 ± 0.003 ^g^	0.020 ± 0.001 ^a^	0.943
CS/SF-1h	55.986 ± 0.404 ^f^	0.023 ± 0.003 ^a^	0.996	0.712 ± 0.003 ^f^	0.039 ± 0.002 ^c^	0.962
CS/SF-1.5h	39.780 ± 0.368 ^c^	0.038 ± 0.004 ^bc^	0.970	0.598 ± 0.002 ^e^	0.021 ± 0.001 ^a^	0.944
CS/SF-2h	33.532 ± 0.147 ^c^	0.029 ± 0.002 ^b^	0.945	0.479 ± 0.002 ^b^	0.033 ± 0.002 ^b^	0.956
CS/SF-4h	30.660 ± 0.186 ^b^	0.022 ± 0.003 ^a^	0.929	0.501 ± 0.004 ^c^	0.022 ± 0.003 ^a^	0.978
CS/SF-8h	26.745 ± 0.306 ^a^	0.037 ± 0.005 ^bc^	0.905	0.456 ± 0.002 ^a^	0.018 ± 0.001 ^a^	0.924
SFs	9.621 ± 0.233 ^a^	0.078 ± 0.010 ^e^	0.911	0.219 ± 0.002 ^a^	0.015 ± 0.004 ^a^	0.905
CS/SF-5%	32.646 ± 0.640 ^b^	0.066 ± 0.008 ^d^	0.922	0.479 ± 0.002 ^b^	0.033 ± 0.002 ^b^	0.956
CS/SF-10%	39.780 ± 0.368 ^c^	0.038 ± 0.004 ^b^	0.970	0.514 ± 0.003 ^c^	0.018 ± 0.002 ^a^	0.994
CS/SF-15%	55.986 ± 0.404 ^e^	0.023 ± 0.003 ^a^	0.996	0.712 ± 0.003 ^d^	0.039 ± 0.002 ^c^	0.962
CS/SF-20%	52.243 ± 0.386 ^d^	0.048 ± 0.003 ^c^	0.943	0.925 ± 0.004 ^e^	0.017 ± 0.002 ^a^	0.950

Values represent the mean ± standard deviation. ^a–^^g^ Within a row, means with different superscript letters differ significantly (*p* < 0.05).

**Table 5:** The parameters of Burger’s models for the CS/SFs with different ball grinding times and addition amounts.

Sample	*E_M_* (×10^8^ MPa)	*E_K_* (MPa)	*τ* (s)	*η_M_* (MPa·s)	*R* ^2^	*ε*′(*∞*) (×10^−3^ s^−1^)
CS/SF-0h	0.298 ± 0.526 ^a^	0.739 ± 0.047 ^a^	0.163 ± 0.019 ^b c^	9.952 ± 1.164 ^a^	0.982	101.411 ± 11.943 ^e^
CS/SF-0.5h	2.058 ± 0.031 ^c^	2.192 ± 0.129 ^bc^	0.398 ± 0.072 ^d^	103.538 ± 13.847 ^d^	0.956	33.240 ± 0.447 ^d^
CS/SF-1h	16.120 ± 0.079 ^g^	4.873 ± 0.313 ^e^	0.584 ± 0.069 ^e^	1607.628 ± 9.522 ^f^	0.988	0.961 ± 0.107 ^a^
CS/SF-1.5h	3.606 ± 0.011 ^f^	2.829 ± 0.110 ^d^	0.151 ± 0.008 ^b^	1041.054 ± 4.021 ^e^	0.982	11.000 ± 0.669 ^b^
CS/SF-2h	2.286 ± 0.440 ^e^	2.431 ± 0.128 ^c^	0.153 ± 0.009 ^b^	87.911 ± 2.236 ^c^	0.983	11.380 ± 0.291 ^b^
CS/SF-4h	2.172 ± 0.100 ^d^	1.855 ± 0.059 ^b^	0.220 ± 0.039 ^c^	68.579 ± 0.283 ^b^	0.950	14.582 ± 0.062 ^c^
CS/SF-8h	0.608 ± 0.018 ^b^	1.720 ± 0.120 ^b^	0.121 ± 0.017 ^a^	65.236 ± 8.600 ^b^	0.978	15.510 ± 2.062 ^c^
SFs	1.127 ± 0.031 ^a^	1.007 ± 0.093 ^a^	12.940 ± 0.920 ^a^	29.065 ± 3.222 ^a^	0.982	2.713 ± 0.038 ^e^
CS/SF-5%	1.265 ± 0.021 ^b^	1.512 ± 0.070 ^b^	24.273 ± 0.544 ^c^	52.541 ± 3.545 ^b^	0.989	1.532 ± 0.032 ^d^
CS/SF-10%	1.374 ± 0.003 ^c^	2.017 ± 0.102 ^c^	20.372 ± 0.852 ^b^	117.328 ± 16.490 ^d^	0.991	0.742 ± 0.097 ^b^
CS/SF-15%	1.752 ± 0.037 ^e^	3.742 ± 0.111 ^d^	20.879 ± 1.307 ^b^	428.140 ± 31.108 ^e^	0.983	0.199 ± 0.017 ^a^
CS/SF-20%	1.659 ± 0.043 ^d^	2.077 ± 0.252 ^c^	23.611 ± 0.367 ^c^	93.358 ± 7.062 ^c^	0.992	0.865 ± 0.033 ^c^

Values represent the mean ± standard deviation. ^a–^^g^ Within a row, the means with different superscript letters differ significantly (*p* < 0.05).
